# Quality of reporting of systematic reviews published in “evidence-based” Chinese journals

**DOI:** 10.1186/2046-4053-3-58

**Published:** 2014-06-07

**Authors:** Jin-long Li, Long Ge, Ji-chun Ma, Qiao-ling Zeng, Lu Yao, Ni An, Jie-xian Ding, Yu-hong Gan, Jin-hui Tian

**Affiliations:** 1 Evidence-Based Medicine Center of Lanzhou University, Lanzhou 730000, Gansu, China; 2 The First Clinical Medicine College of Lanzhou University, Lanzhou 730000, China; 3 The Second Clinical Medicine College of Lanzhou University, Lanzhou 730000, China

**Keywords:** Chinese journal, Evidence-based, Meta-analyses, Quality of reporting, Systematic reviews

## Abstract

**Background:**

The number of systematic reviews (SRs)/meta-analyses (MAs) has increased dramatically in China over the past decades. However, evaluation of quality of reporting of systematic reviews published has not been undertaken. The objective of this study is to evaluate the quality of reporting of SRs/MAs assessing efficacy and/or harms of clinical interventions published in “evidence-based” Chinese journals.

**Methods:**

Web-based database searches were conducted for the *Chinese Journal of Evidence-based Medicine*, the *Journal of Evidence-Based Medicine*, the *Chinese Journal of Evidence Based Pediatrics*, and the *Chinese Journal of Evidence-Based Cardiovascular Medicine*. SRs/MAs assessing efficacy and/or harms of clinical interventions were included. The cut-off was December 31st 2011. The PRISMA statement was applied to assess the quality of reporting. Each item was assessed as follows: ‘Yes’ for total compliance, scored ‘1’; ‘partial’ for partial compliance, scored ‘0.5’; and ‘No’ for non-compliance, scored ‘0’. The review was considered to have major flaws if it received a total score of ≤15.0, minor flaws if it received a total score of 15.5 to 21.0, and minimal flaws if it received a total score 21.5 to 27.0. Odds ratios were used for binary variables, and the mean difference was used for continuous variables. Analyses were performed using RevMan 5.0 software.

**Results:**

Overall, 487 SRs/MAs were identified and assessed. The included reviews had medium quality with minor flaws based on PRISMA total scores (range: 8.5–26.0; mean: 19.6 ± 3.3). The stratified analysis showed that SRs/MAs with more than 3 authors, from a university, hospital + university cooperation, multiple affiliations (≥2), and funding have significantly higher quality of reporting of SRs/MAs; 58% of the included reviews were considered to have minor flaws (total score of 15.6 to 21.0). Only 9.6% of reviews were considered to have major flaws. Specific areas needing improvement in reporting include the abstract, protocol and registration, and characteristics of the search.

**Conclusions:**

The reporting of SRs published in “evidence-based” Chinese journals is poor and needs to be improved in order for reviews to be useful. SR authors should use the PRISMA checklist to ensure complete and accurate accounts of their SRs.

## Background

Clinicians refer to systematic reviews (SRs)/meta-analyses (MAs) to keep up to date with developments in their field. High quality SRs/MAs of randomized controlled trials can provide the best evidence [1]. In order to assess the quality of reporting of SRs/MAs, complete, clear, and transparent information with regards to the design and conduct of the SRs/MAs are required; however, to date, this information it is not optimal and the low quality of reporting diminishes the value of SRs/MAs for clinicians, policy makers, and other users [2].

In order to improve the quality of reporting of SRs/MAs, an international group of experienced authors and methodologists developed the Preferred Reporting Items of Systematic Reviews and Meta-Analyses (PRISMA) [3], which itself was a successor to the original QUOROM guidelines [4]. It contains a 27-item checklist and a 4-phase flow diagram. The checklist includes items deemed essential for transparent reporting of a SR [2].

A few studies have examined the reporting quality of SRs/MAs in various fields, and found that the reporting quality of SRs/MAs was poor with many flaws [5-8]. Recently, the concept of evidence-based medicine has widely spread across China through the *Chinese Journal of Evidence-based Medicine* (*CJEBM*), the *Journal of Evidence-Based Medicine* (*JEBM*), the *Chinese Journal of Evidence-Based Pediatrics* (*CJEBP*), and the *Chinese Journal of Evidence-Based Cardiovascular Medicine* (*EBCVM*) [9]. In this study, we sought to determine the degree to which studies published in these four journals met the PRISMA criteria for reporting, whether it had improved after publication of the PRISMA statement, and factors associated with good reporting.

## Methods

Sample collection, selection, data extraction, and quality assessment were performed independently by two trained reviewers; disagreements between reviewers were resolved through consensus or by consulting a third expert adjudicator.

### Selection of studies

An inclusion criterion was that the words SR and/or MA were stated in the title of studies. Using this criterion, we searched the online databases of the *CJEBM* (http://www.cjebm.org.cn, 2001 to December 31st, 2011), the *JEBM* (http://www.jebm.cn, 2001 to December 31st, 2011), the *CJEBP* (http://www.cjebp.net, 2006 to December 31st, 2011) and the *EBCVM* (http://www.ebcvm.org, 2008 to December 31st, 2011) for SRs and MAs. Only reviews assessing the safety and/or efficacy of clinical interventions were included. A total of 487 SRs and MAs on interventions were included after reading the title, abstract, and full-text.

### Data extraction

We established a spreadsheet using Microsoft Excel 2003. In addition to the items in PRISMA, the following data were extracted from each study: i) title; ii) first author name; iii) the name of the journal; iv) the number of author(s); v) year of publication; vi) the authors’ affiliations (hospital or university) and the number of affiliations; vii) financial support; viii) categories of diseases (ICD-10) [10]; and ix) the number of included studies.

### Statistical analysis

PRISMA was used to evaluate the adherence of review articles to scientific principles. The tool covers seven modules with 27-items: title, abstract, introduction, methods, results, discussion, and funding. Each of the items were assessed as follows: ‘Yes’ for total compliance, scored ‘1’; ‘partial’ for partial compliance, scored ‘0.5’; and ‘No’ for non-compliance, scored ‘0’; with a total maximum score ‘27’. The review was considered to have major flaws if it received a total score of ≤15.0, minor flaws if it received a total score of 15.5 to 21.0, and minimal flaws if it received a total score 21.5 to 27.0 [11].

The percentage of reports that met each criterion was determined and tabulated. Data on each item was presented as counts and percentages. The odds ratio (OR), 95% CI, mean, standard deviation (SD), and mean difference (MD) were used as the summary statistics for stratified comparisons with RevMan software version 5.0. Statistical significance was set at *P* ≤0.05.

### Stratified analysis

We assessed the following variables as potentially associated with differences in the quality of reporting: publication time (≤2009 vs. ≥2010), the number of authors (≤2 vs. ≥3), the authors’ affiliations (hospital vs. university), the number of affiliations (1 vs. ≥2), and funding sources (funding vs. non-funding) [12].

To assess whether publication of the PRISMA statement was associated with improved reporting of certain items, we compared overall compliance in each PRISMA item using the *t*-test (≤2009 vs. ≥2010).

## Results

### Description of included reviews

Four hundred and eighty seven studies were included for evaluation; 77.8% (379/487) of SRs/MAs were published in *CJEBM*, 14.4% (70/487) of SRs were published in *JEBM*, 6.2% (30/487) of SRs were published in *CJEBP*, and *EBCVM* published 1.6% (8/487) of SRs/MAs. The first SR was published in 2001, and the total number increased to 89 in 2009. Twenty categories of diseases were involved according to ICD-10 and the most common conditions studied were neoplasms (17.4%, 83/487). The reviews included a median of four authors (range: 1–12) and 87.7% (427/487) of the studies were written by ≥3 authors; almost half (43.3%) of the reviews were written by clinicians. Financial support was obtained by 25% (122/487) of the reviews. The reviews included a median of 8 randomized controlled trials (range: 1–129).

### The overall reporting quality of included reviews

**Figure 1:**
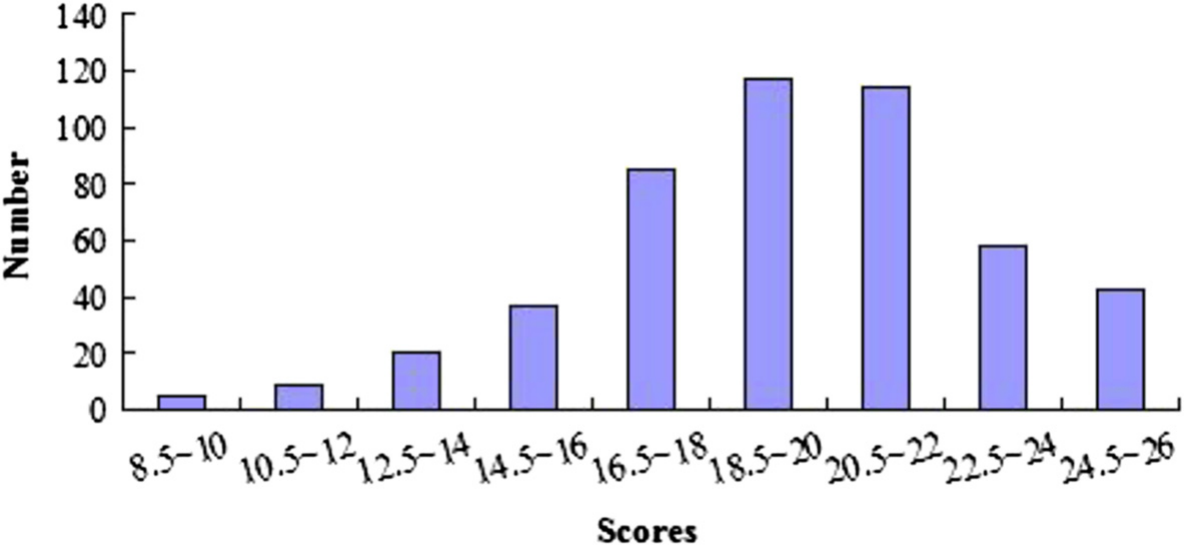
The overall scores for PRISMA.

Overall, the compliance with PRISMA was poor, none of the included reviews fulfilled all 27-items of PRISMA. Table 1 shows that the weakest areas within the included SRs/MAs were the reporting of protocol and registration information (item 5, 0.4%), risk of bias across studies (item 22, 22.4%), additional analyses (item 23, 29.2%), and funding sources (item 27, 24.4%). The reasons for particularly low reporting for item 5 may be that there is no existing register for SRs/MAs in China, and the relatively rare publication of SR/MA protocols. Some PRISMA items were reported in >90% of SRs/MAs, including title (item 1, 97.7%), eligibility criteria (item 6, 91.8%), information sources (item 7, 98.6%), summary measures (item 13, 91.2%), synthesis of results (**methods section**) SRs/MAs(item 14, 93.0%), study characteristics (item 18, 91.4%), results of individual studies (item 20, 90.8%), synthesis of results (**results section**) SRs/MAs(item 21, 90.8%), and summary of evidence (item 24, 90.3%). The overall quality scores are shown in Figure 1. The range and mean ± SD of overall quality score for included SRs/MAs was 8.5 to 26.0 and 19.6 ± 3.3, respectively; 47 (9.6%) studies had major flaws (an overall score of ≥15), 284 (58.3%) had minor flaws (an overall score of 15.5 to 21.0), and 156 (32%) were considered to have minimal flaws (an overall score of 21.5 to 27.0).

**Table 1:** The results of reporting quality assessment (n = 487)

**PRISMA items**		**Yes**		**Partial**		**No**	
		**n (%)**	**95% CI**	**n (%)**	**95% CI**	**n (%)**	**95% CI**
**Title**	**1. Title**	476 (98)	96–99	1 (0)	1–1	10 (2)	1–4
**Abstract**	**2. Structured summary**	256 (53)	48–57	226 (46)	42–51	5 (1)	0–2
**Introduction**	**3. Rational**	429 (88)	85–91	55 (11)	9–14	3 (1)	0–2
	**4. Objective**	335 (69)	65–73	143 (29)	26–34	9 (2)	1–4
**Methods**	**5. Protocol and registration**	2 (0)	0–2	58 (12)	9–15	427 (88)	85–90
	**6. Eligibility criteria**	447 (92)	89–94	39 (8)	6–11	1 (0)	1–1
	**7. Information sources**	480 (99)	97–100	6 (1)	0–23	1 (0)	0–1
	**8. Search**	263 (54)	50–59	182 (37)	33–41	42 (9)	6–12
	**9. Study selection**	241 (50)	45–54	72 (15)	12–18	174 (36)	32–40
	**10. Data collection process**	334 (69)	64–73	45 (9)	7–12	108 (22)	19–26
	**11. Data items**	203 (42)	37–46	25 (5)	4–8	259 (53)	49–58
	**12. Risk of bias in individual studies**	436 (90)	87–92	17 (4)	2–6	34 (7)	5–10
	**13. Summary measures**	444 (91)	88–93	7 (1)	0–3	36 (7)	5–10
	**14. Synthesis of results**	453 (93)	90–95	7 (1)	0–3	27 (6)	4–8
	**15. Risk of bias across studies**	161 (33)	29–37	74 (15)	12–19	252 (52)	47–56
	**16. Additional analyses**	253 (52)	48–56	44 (9)	7–12	190 (39)	35–43
**Results**	**17. Study selection**	356 (73)	69–77	70 (14)	12–18	61 (13)	10–16
	**18. Study characteristics**	445 (91)	89–94	21 (4)	3–7	21 (4)	3–7
	**19. Risk of bias with studies**	423 (87)	84–90	31 (6)	5–9	33 (7)	5–9
	**20. Results of individual studies**	442 (91)	88–93	24 (5)	3–7	21 (4)	3–7
	**21. Synthesis of results**	442 (91)	88–93	23 (5)	3–7	22 (5)	3–7
	**22. Risk of bias across studies**	109 (22)	19–26	94 (19)	16–23	284 (58)	54–63
	**23. Additional analyses**	142 (29)	25–33	56 (11)	9–15	289 (59)	55–64
**Discussion**	**24. Summary of evidence**	440 (90)	87–93	38 (8)	6–11	9 (2)	1–4
	**25. Limitations**	385 (79)	75–83	42 (9)	6–12	60 (12)	10–16
	**26. Conclusions**	394 (81)	77–84	85 (18)	14–21	8 (2)	0–3
**Funding**	**27. Funding**	119 (24)	21–28	41 (8)	6–11	327 (67)	63–71
**Total score**	**Scope**	8.5–26.0					
	**±SD**	19.60 ± 3.33					

### Did the quality of SRs/MAs improve post-PRISMA?

**Figure 2:**
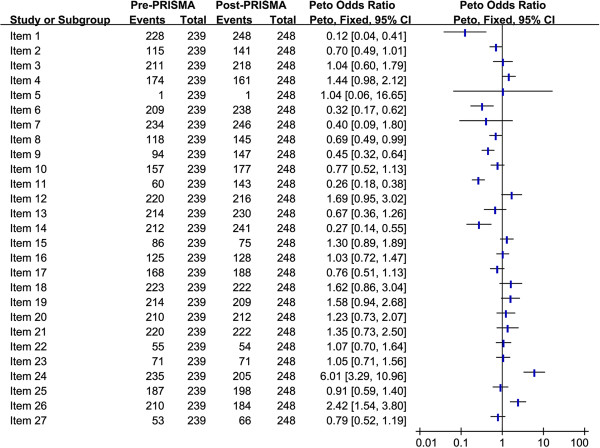
Comparison of pre-PRISMA and post-PRISMA periods for each PRISMA item.

To investigate whether the publication of the PRISMA statement was associated with an improvement in reporting for certain items (pre-PRISMA vs. post-PRISMA), the period ≤2009 was compared with ≥2010 in each of the PRISMA statement. Figure 2 shows that, following publication of the PRISMA statement, the quality of reporting improved; the difference was statistically significant for following items: item 1 (*P* = 0.0006), item 6 (*P* = 0.0006), item 8 (*P* = 0.04), item 9 (*P* <0.0001), item 11 (*P* <0.00001), and item 14 (*P* = 0.0002).

### Stratified analysis

**Figure 3:**
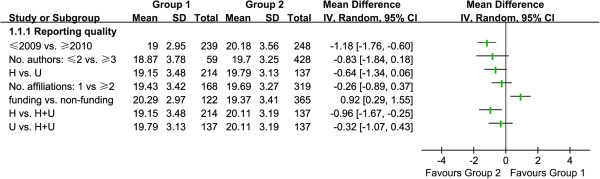
Stratified analysis of included SRs/MAs.

To assess possible factors associated with reporting quality, we analyzed the publication time (≤2009 vs. ≥2010), the number of authors (≤2 vs. ≥3), the affiliation of authors (hospital vs. university), the number of affiliations (1 vs. ≥2), and funding sources (funding vs. non-funding), as shown in Figure 3. The overall quality scores of SRs/MAs published post-PRISMA was significantly different, with a mean difference of –1.18 (95% CI: –1.76 to –0.60). The overall quality score of SRs/MAs written by ≥3 authors showed no statistically significant difference compared to by 1 to 2 authors (MD: –0.8 [95% CI: –1.8 to 0.2]). SRs conducted within universities (MD: –0.6 [95% CI: –1.3 to 0.1]) or hospital + university cooperation (MD: –0.9 [95% CI: –1.7 to –0.3]) were better reported than those conducted outside from hospitals. SRs conducted by authors with more than two affiliations were better than one affiliation (MD: –0.3 [95% CI: –0.9 to 0.4]), and funded reports demonstrated a statistically significant difference when compared with non-funded reports (MD: 0.9 [95% CI: 0.3 to 1.6]).

### Comparison of overall PRISMA scores in the four journals assessed

Table 2 shows that the overall quality score of SRs/MAs published in the four journals demonstrated minor flaws (58.3%), and we found that the quality of reporting of SRs/MAs published in *CJEBP* (21.8 ± 1.5) was better when compared with the remaining journals (19.5 ± 3.4) (MD: 2.3 [95% CI: 1.7 to 2.9], *P* <0.00001).

**Table 2:** Comparison of overall PRISMA scores in the four assessed journals [n (%)]

	**CJEBM (n = 379)**	**JEBM (n = 70)**	**CJEBP (n = 30)**	**EBCVM (n = 8)**	**Total (n = 487)**
**≤15**	25 (7)	22 (31)	0	0	47 (10)
**~21**	226 (60)	43 (61)	10 (33)	5 (63)	284 (58)
**~27**	128 (34)	5 (7)	20 (67)	3 (38)	156 (32)
**Range**	8.5–26	8.5–23	18.5–24.5	18–21.5	8.5–26
**Mean ± SD**	19.97 ± 3.15	16.60 ± 3.22	21.77 ± 1.52	20.19 ± 1.41	19.60 ± 3.33

## Discussion

The number of SRs/MAs published in China has been accumulating at an increasing rate in recent years. However, the results of our study indicate that the quality of the SRs/MAs is not optimal.

The best reported items (addressed completely >90.0%) included identification of a SR/MA by its title, eligibility criteria, information sources (such as the database or key words searched), summary measures, synthesis of results (methods section), study characteristics, results of individual studies (results section), synthesis of results (results section), and summary of evidence (results section). A great deal of improvement is required in the following items (addressed completely 50% to 90%): structured summary, rational, search, data collection process, risk of bias in individual studies (methods section), additional analyses (methods section), study selection, risk of bias with studies (results section), limitations, and conclusions. Furthermore, there was seriously flawed reporting (addressed completely 50% or less) in protocol and registration, study selection (methods section), data items (methods section), risk of bias across studies (methods section), risk of bias across studies (results section), additional analyses (results section), and funding sources.

Further, the registration of protocols was rarely performed. We expect that a network of registration for SRs will be established in China, thus facilitating the registration and reporting of protocols. The main reason most studies scored poorly on details of the search strategy was that only 19.7% reviews reported the detailed search strategy; most reviews only reported the databases and key words. Chinese journals should demand that authors provide the detailed search strategy.

## Conclusions

SRs or MAs related to efficacy and safety of clinical interventions published in “evidence-based” Chinese journals had minor flaws. Efforts should be made to improve the reporting of a structured abstract, objectives, protocol and registration, search strategy, data extracted, risk of bias across studies, additional analyses, and funding sources reporting. Meanwhile, SR authors should use the PRISMA checklist to ensure complete and accurate accounts of their SRs.

## Abbreviations

SRs: Systematic reviews; MAs: Meta-analyses; PRISMA: Preferred Reporting Items for Systematic Reviews and Meta-Analyses; CJEBM: Chinese Journal of Evidence-based Medicine; JEBM: Journal of Evidence-Based Medicine; CJEBP: Chinese Journal of Evidence Based Pediatrics; EBCVM: Chinese Journal of Evidence-Based Cardiovascular Medicine; OR: Odds ratio; CI: Confidence interval; SD: Standard deviation; MD: Mean difference.

## Competing interests

The authors declare that they have no competing interest.

## Authors’ contributions

TJH and GL have participated in all study processes, including the concept and design, analysis and interpretation of data, drafting and revising of the manuscript, and approving the manuscript. LJL has participated in analysis and interpretation of data, and written the manuscript. DJX, GYH, YL, and AN have participated in literature search, data extraction, and quality assessment. MJC and ZQL have participated in statistical analysis and drafting and revising of the manuscript. All authors read and approved the final manuscript.
